# Production of drug metabolites by human FMO3 in *Escherichia coli*

**DOI:** 10.1186/s12934-020-01332-1

**Published:** 2020-03-20

**Authors:** Gianluca Catucci, Gianfranco Gilardi, Sheila J. Sadeghi

**Affiliations:** Department of Life Sciences and Systems Biology, University of Torino, Via Accademia Albertina 13, 10123 Turin, Italy

**Keywords:** Human flavin-containing monooxygenase isoform 3, Drug metabolites, Biocatalysis, Whole-cell, Clomiphene, Dasatinib, Tozasertib, GSK5182, Regioselectivity

## Abstract

**Background:**

In the course of drug discovery and development process, sufficient reference standards of drug metabolites are required, especially for preclinical/clinical or new therapeutic drugs. Whole-cell synthesis of drug metabolites is of great interest due to its low cost, low environmental impact and specificity of the enzymatic reaction compared to chemical synthesis. Here, *Escherichia coli* (*E. coli*) JM109 cells over-expressing the recombinant human FMO3 (flavin-containing monooxygenase isoform 3) were used for the conversions of clomiphene, dasatinib, GSK5182 and tozasertib to their corresponding N-oxide metabolites.

**Results:**

The effects of NADPH regeneration, organic solvents as well as C-terminal truncations of human FMO3 were investigated. Under the optimized conditions, in excess of 200 mg/L of N-oxide metabolite of each of the four drugs could be produced by whole-cell catalysis within 24 h. Of these, more than 90% yield conversions were obtained for the N-oxidation of clomiphene and dasatinib. In addition, FMO3 shows high regio-selectivity in metabolizing GSK5182 where only the (Z) isomer is monooxygenated.

**Conclusions:**

The study shows the successful use of human FMO3-based whole-cell as a biocatalyst for the efficient synthesis of drug metabolites including regio-selective reactions involving GSK5182, a new candidate against type 2 diabetes mellitus.

## Background

Human flavin-containing monooxygenase isoform 3 (hFMO3) is the most important non-cytochrome P450 Phase I drug metabolizing enzyme in adult human liver, catalysing the monooxygenation of a wide variety of nucleophilic heteroatom-containing drugs, xenobiotics and dietary compounds [1–3]. In the lengthy process of drug development, reference standards of drug metabolites under investigation are required for validation of their chemical structures as well as pharmacological characterization [4, 5]. Nevertheless, such standards are generally not available in the case of new therapeutic drugs. One option available to pharmaceutical companies is chemical synthesis of the drug metabolites however, the latter solution is very demanding both in terms of time and capital [6]. In addition, although drug metabolites can be directly generated in vitro by enzymes with high selectivity, the synthesis on large scale is challenging due to low catalytic activity and/or protein stability, requirement for the expensive cofactor(s) as well as low expression of membrane-bound enzymes [6]. In this context, the possibility of synthesizing drug metabolites, through a whole-cell system with the inclusion of a human enzyme, becomes extremely attractive due to its many advantages: low cost, cofactor regeneration, stable enzyme, large scale application and uncomplicated downstream processing [5].

**Fig. 1:**
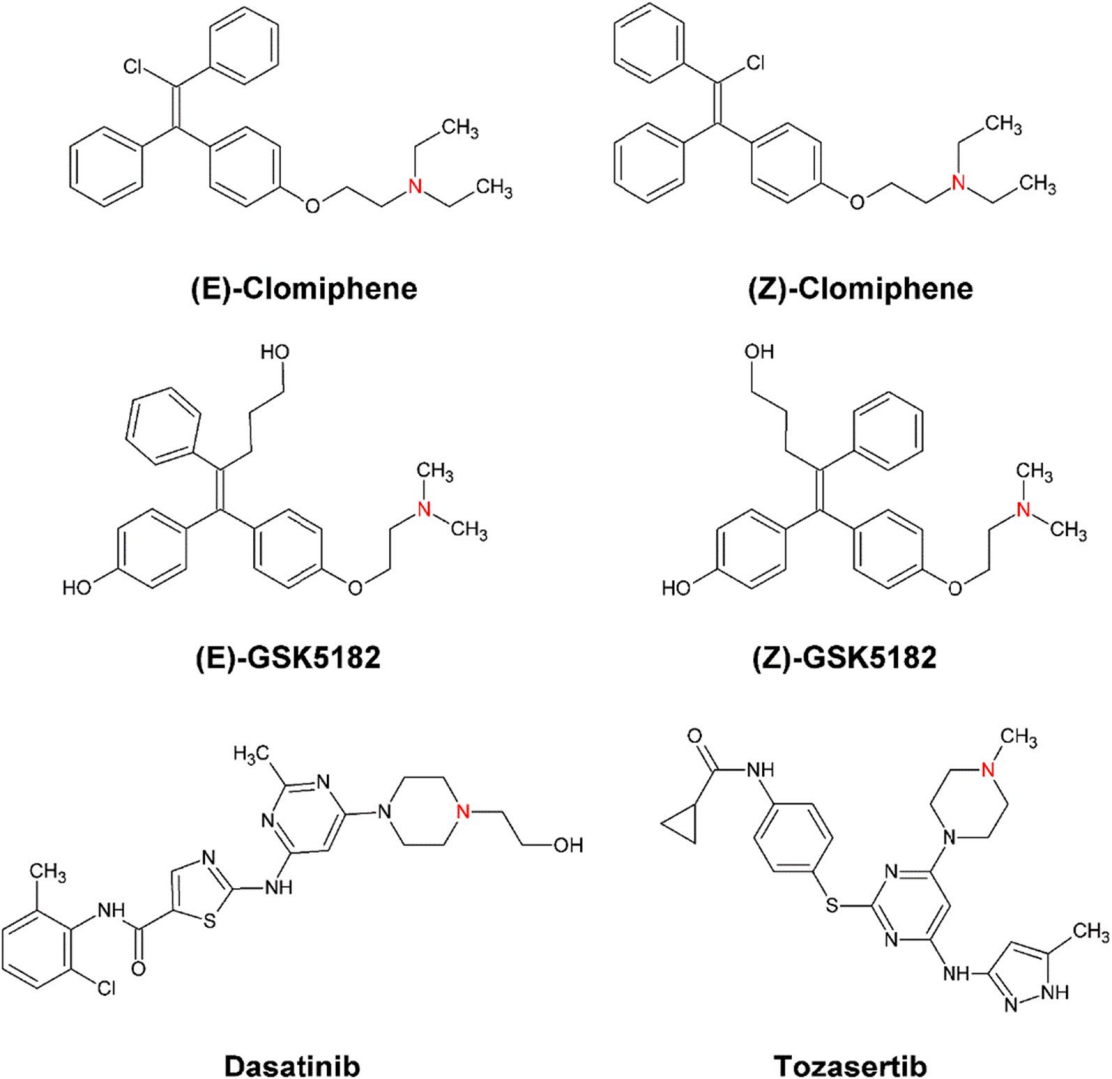
The chemical structures of the four drugs studied in this work including the E- and Z-isomers. The monooxygenation site of each compound is highlighted in red

The main criterion for whole-cell catalysis is the expression of the target enzyme in its functional form. In this paper, human FMO3 over expressed in *Escherichia coli (E. coli)* JM109 [8–10] (using the pJL2 expression vector [7]) was employed for whole-cell catalysis experiments with four different drug substrates; clomiphene, dasatinib, GSK5182 and tozasertib. The chemical structures of these drug substrates and the site of their monooxygenation are shown in Fig. 1. Clomiphene is the most commonly used non-steroidal fertility medication for woman [11, 12] and has recently been shown to be metabolized by human FMO3 [13]. Dasatinib, a multi-targeted kinase inhibitor is used for the treatment of chronic myelogenous leukemia and acute lymphoblastic leukemia [14] and FMO3 been shown to be involved in its metabolism [14]. GSK5182, a highly selective inverse agonist of estrogen-related receptor γ, is currently under preclinical investigation as a new anti-diabetic agent for type 2 diabetes mellitus [15]. N-demethylation and hydroxylation of this drug are carried out by cytochrome P450s whereas FMO3 contributes to the GSK5182 N-oxidation [16]. Tozasertib (VX-680, MK0457), a pan-aurora kinase inhibitor, is under evaluation as an anticancer drug. It is metabolised in the human liver via two major pathways one of which involves the FMO3-mediated N-oxidation [17, 18].

In this work, the effect of different chemicals on NADPH regeneration as well as organic solvents, on the overall conversion of the high value metabolites of the above-mentioned drugs was investigated. Moreover, hFMO3 is anchored to the ER membranes through its C-terminus and therefore the effect of several truncations at the C-terminal on its whole-cell over expression and activity were also investigated.

## Results and discussion

### Compounds promoting NADPH formation

In the first set of experiments and in order to carry out the optimization of the different parameters involved in whole-cell biotransformations, sufficient biomass was produced as outlined in methods and materials with the overexpression of human FMO3 in *E. coli* JM109. One of the most important bottlenecks in using flavoenzymes for the production of drug metabolites is their reliance on the expensive NADPH cofactor and therefore this parameter was tackled first. For economically efficient utilisation of human FMO3, the consumed reduced equivalents have to be regenerated. This can be done by the addition of one or more enzymes to the reaction but a much simpler method is the use of whole-cells. Previous studies have indicated that using a whole-cell system, the naturally existing cellular metabolic pathways can be exploited for the regeneration of this cofactor [19]. Moreover, in the specific case of FMO enzymes, Hanlon et al. [20] have demonstrated that addition of both citrate and NADP^+^ to the whole-cell is necessary for full activity of these enzymes. We therefore proceeded with testing citrate and NADP^+^ in our experimental set up which includes both a different bacterial strain (*E. coli* JM109 versus BL21 DE3 GOLD) and FMO clone (pJL2 [7] versus pEamTA).

**Fig. 2:**
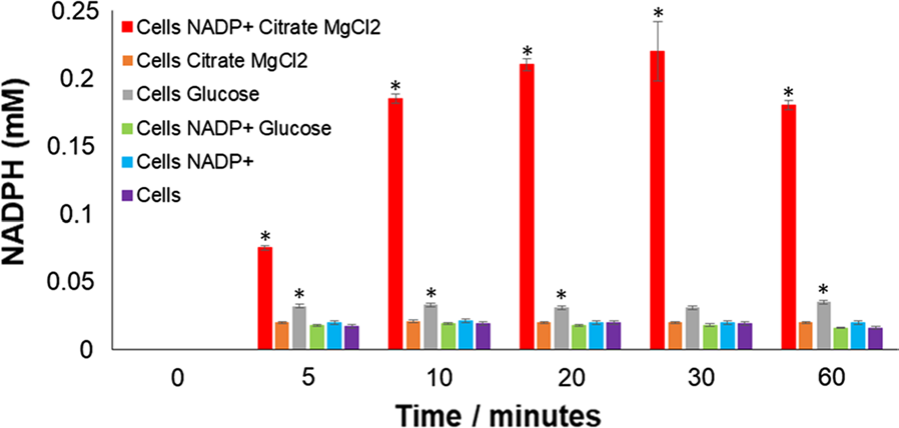
NADPH regeneration measured spectrophotometrically at 340 nm using recombinant *E. coli* JM109 cells in the presence of different combinations of 0.3 mM NADP^+^, 50 mM citrate/10 mM MgCl_2_ (without any substrate) and glucose (50 mM). Experiments were performed three times and the error bars represent the standard deviation obtained from the three replicates. *P  <  0.001 compared to control (cells alone with no other additions) at the same time point; Statistical analyses were carried out using One-way ANOVA followed by Student–Newman–Keuls post hoc test

Figure 2 summaries the NADPH regeneration experiments carried out with the *E. coli* JM109 cells. As can be seen in the figure, a faster regeneration rate was obtained in the presence of both NADP^+^ and citrate/MgCl_2_ compared with either NADP^+^ or citrate/MgCl_2_ alone, as also demonstrated by Hanlon et al. [20]. We also tested the addition of glucose and although an improvement in NADPH regeneration was observed (Fig. 2, asterisks), it was not as pronounced as that already observed in the presence of NADP^+^ and citrate/MgCl_2_. Therefore, all subsequent whole-cell catalyses were carried out in the presence of both NADP^+^ and citrate/MgCl_2_.

For the whole-cell catalyses experiments two controls were also carried out to confirm that only cells expressing the hFMO3 protein were responsible for the products observed: (a) conversion using non-transformed *E. coli* JM109 cells resulted in no detectable product within 24 h; (b) conversion using the supernatant of the pelleted cells also resulted in no detectable product after 24 h, proving the integrity of the bacterial cells (data not shown).

The second parameter to be considered was the amount of drug to be employed in the whole-cell reactions. In biocatalysis, it has been shown that depending on the substrate concentration one can get either higher product or lower conversions due to product inhibitory effects. Like other drug metabolizing enzymes, majority of known substrates of hFMO3 are hydrophobic compounds with low solubility in aqueous buffers. The latter observation leads to a limitation in the enzymatic efficiency of this drug metabolizing enzyme. One solution to overcome this problem is the use of water miscible organic solvents with higher polarity index to develop mono- or biphasic media for efficient biocatalysis [21].

To this end, ten different solvents were tested for the whole-cell production of tamoxifen N-oxide within 5 h as described in “Methods” section. Tamoxifen, a breast cancer therapeutics and known substrate of human FMO [8, 22], is soluble in organic solvents but insoluble in water (solubility is < 0.01%, 20 °C). Within our lab we routinely employ this drug as a marker substrate for the activity of purified human FMO enzymes [8] and therefore it was used to investigate the effect of different solvents on conversion yields. Nonetheless, the results obtained with the selected solvents demonstrated not only a decrease in conversion yields but also no correlation between the polarity index of the solvents and the observed percentage conversions (Additional file 1: Table S1).

### Effect of human FMO3 truncations on whole-cell catalysis

Another factor to be considered was the expression level of human FMO3 in the bacterial system, as higher expression levels in whole-cells would most probably improve the product titre. Human FMO3 is a membrane associated protein and difficult to overexpress in bacterial cells as many other membrane-bound proteins [23]. In order to improve the solubility of human FMO3, we had previously reported the construction of three different C-terminal truncations of this protein with the deletion of 16, 27 and 39 amino acids by site-directed mutagenesis resulting in variants 516X, 505X and 493X, respectively [24]. Whereas the majority of the full-length human FMO3 was found in the membrane-bound fraction, the two more conservative truncations, 516X and 505X, although not completely detached from the membrane were found in the cytosolic fraction. Finally, for the most extreme truncation, 493X, was mainly cytosolic as a consequence of the higher solubility of the resulting truncated hFMO3 protein.

The above improvement of the cytosolic expression of the truncated variants of human FMO3 was initially taken as a positive result, thought to favour protein overexpression in *E. coli* and as a result the conversion yields of the metabolites. In order to prove the latter hypothesis, the three truncated FMO3 proteins were expressed in *E. coli* and used for whole-cell catalysis and production of the test metabolite, tamoxifen N-oxide. The reactions were performed at 37 °C for 24 h and the results obtained are summarized in Table 1. As can be seen in the table and contrary to our hypothesis, the C-terminally truncated enzymes demonstrated a drastic decline in biocatalysis of tamoxifen when compared to the membrane-bound human FMO3. More in depth studies are required to ascertain as to why the more soluble enzyme is not favoured in the whole cell biocatalysis. However, similar results were reported by Winker’s group [5] when they attempted to solubilize human FMO2 and use it as a whole-cell catalyst. They concluded that the deletion of several amino acids at the C-terminal of FMO2 might have affected the protein’s folding and/or stability.

**Table 1:** Effect of the different C-terminal truncations of human FMO3 on the conversion of the test substrate, tamoxifen, compared to the full-length protein

FMO3	Conversion (%)
Full-length	78.0 ± 2.0
516X	4.0 ± 0.2
505X	10.0 ± 0.4
493X	1.0 ± 0.1

The experiments were carried out in triplicates. Data represent the mean (± standard deviation, SD) of the three independent experiments

### Whole-cell catalysis of clomiphene, tozasertib, dasatinib and GSK5182

**Fig. 3:**
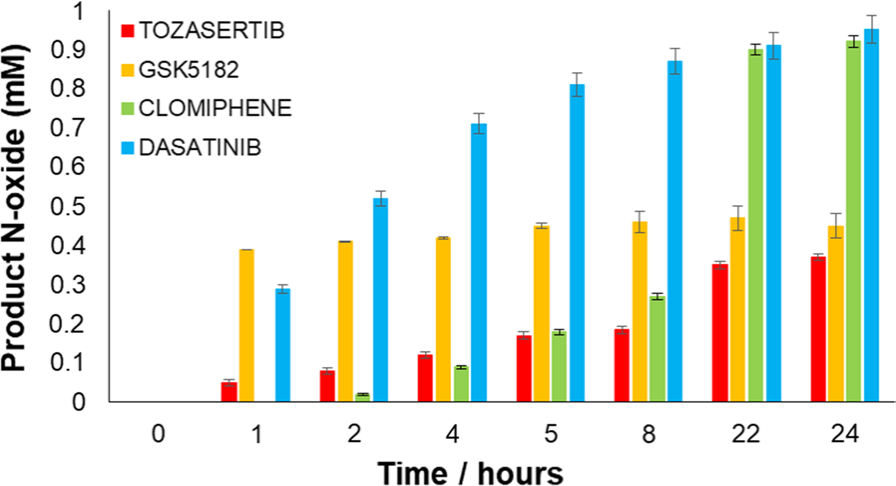
Human FMO3-based whole-cell conversion of GSK5182, clomiphene, tozasertib and dasatinib to their corresponding N-oxide metabolites within a 24-h period. Experiments were performed in triplicates with the error bars representing the standard deviation. No metabolite was detected in the control experiments within 24 h (non-transformed *E. coli* JM109 cells in the presence of the four different substrates)

The whole-cell set up was finally employed for the production of the human FMO3 drug metabolites as described in “Methods” section. The data obtained for the 4 different drugs are shown in Fig. 3 and summarized in Table 2. In the case of dasatinib N-oxygenation, more than 90% of the parent drug was transformed to the metabolite within 24 h. Dasatinib N-oxide was identified and confirmed by LC–MS (Additional file 1: Fig. S1).

**Table 2:** Conversion yields for different substrates of hFMO3 using whole-cell catalysis at 37 °C within 24 h

Substrate	N-oxide product (mg/L)	Conversion (%)
Clomiphene	392.4 ± 1.7	93.0 ± 0.4
Dasatinib	499.0 ± 3.5	99.0 ± 0.7
GSK5182	208.8 ± 1.5	98.4 ± 0.7
Tozasertib	201.8 ± 2.9	42.0 ± 0.6

Data represent the mean (± standard deviation, SD) of three independent experiments

Commercial clomiphene is a mixture of two geometric isomers, (E)- and (Z)-clomiphene [13]. The results demonstrate that both of these two isomers can be converted to the N-oxide product by FMO3-based whole-cell biocatalysis, with an overall conversion yield of 93% (Table 2). In this case, the human FMO3 shows no selectivity in its activity. The identification of the N-oxide products of clomiphene have been previously reported by our group [16].

**Fig. 4:**
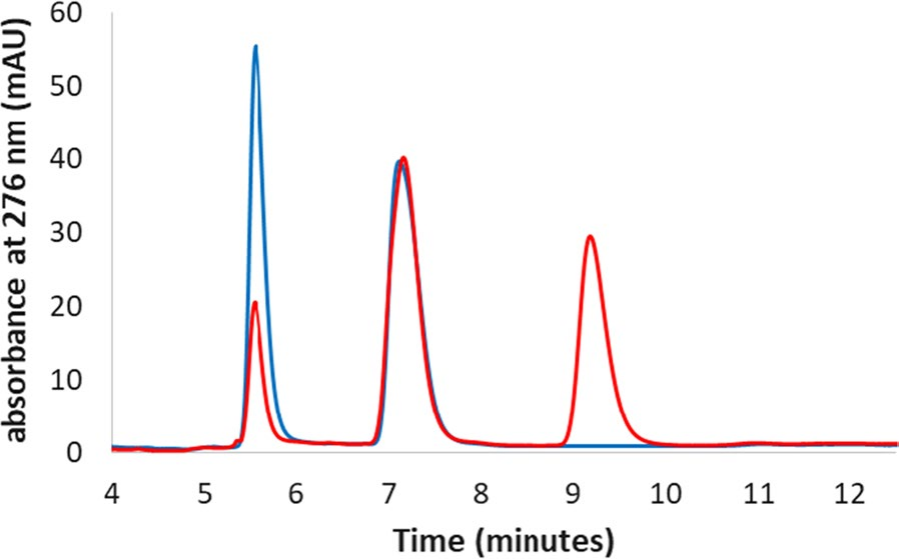
The superimposed HPLC chromatograms of GSK5182 (blue trace) and its conversion to its N-oxide by human FMO3 (red trace). The enzyme shows regioselectivity only metabolizing the Z-isomer (retention time of 5.5 min), while the E-isomer is untouched (retention time of 7.5 min). The N-oxide product (retention time of 9.3 min) was identified by LC–MS

A more interesting result was obtained for GSK5182 where hFMO3 shows high regio-selectivity by converting only one of the two isomers (parent drug sold with an isomer ratio of 1:1), the Z-isomer, to its corresponding N-oxide product as shown in Fig. 4. There are currently no commercially available standards and therefore the N-oxide product identification was performed by LC–MS [16]. An overall conversion of 98.4% was obtained for this drug which corresponds to the maximum theoretical conversion yield (Fig. 4 and Table 2).

Out of the four drugs tested, the lowest conversion yield was obtained with tozasertib. The N-oxide product had also been identified by LC–MS and reported by our group previously [25]. In this case, the biotransformation to the N-oxide metabolite reached only 42%. One possible explanation for this observation is the lower solubility of the tozasertib parent drug compared to the other tested drugs [18].

## Conclusions

Many approved pharmaceutical drugs and drug candidates contain nitrogen, a soft nucleophile that is metabolized by human FMO3. The data presented demonstrate the successful use of recombinant human FMO3 in a whole-cell set up as a biocatalyst for drug metabolite synthesis. This whole-cell system with the specified cofactor recycling of human FMO3, allowed for the production of high value drug metabolites from clomiphene, dasatinib and GSK5182, with conversion yields ranging from 93.0 to 99.0%, corresponding to 200 mg of each product per liter of cells within a 24-h period. Tozasertib conversion reached only around 40%. These drugs span a whole range of chemical structures as well as varied functionality i.e. from promising diabetes drug to cancer therapeutics. More importantly, the regio-selectivity of this human enzyme is also demonstrated in its reaction with GSK5182, currently available only as a mixture of the two isomers.

Finally, this whole-cell set up, combination of recombinant *E. coli* JM109 with human FMO3, is a promising “green” and cost-effective tool for generation of reference drug metabolite standards not only for drug metabolism studies but also for use during drug discovery and development process.

## Methods

### Chemicals

Acetonitrile, ampicillin, flavin adenine dinucleotide disodium salt hydrate (FAD), benzydamine hydrochloride, tamoxifen, clomiphene citrate salt, NADPH, Isopropyl-ß-D-thiogalactopyranoside (IPTG), IGEPAL (octylphenoxy poly(ethyleneoxy)ethanol) and potassium phosphate were purchased from Sigma-Aldrich.

The drugs under study were purchased from different sources, clomiphene from DBA Italia (Italy); tozasertib from Aurogene (Rome, Italy); dasatinib from Selleck Chemicals (Houston, USA) and GSK5182 (4-[1-[4-[2-(Dimethylamino)ethoxy]phenyl]-5-hydroxy-2-phenylpent-1-enyl]phenol) (with isomer ratio of 1:1, E:Z) from Glaxosmithkline (UK).

### Whole-cell preparation

Human FMO3 (with a C-terminal His tag) inserted into pJL2 was expressed in *E. coli* JM109 as previously reported [8, 24] and exploited for whole-cell catalysis. Non-transformed *E. coli* JM109 cells were used as the negative control. 4 L cultures of the human FMO3 in terrific broth (eight 2L Pyrex glass Erlenmeyer flasks each with 500 mL media) were inoculated with an overnight inoculum of the recombinant *E. coli* JM109 to generate the necessary biomass for whole-cell catalysis studies. The cells were incubated at 37 °C with 200 rpm agitation until an OD_600_ of 0.8 was reached before induction with 1 mM IPTG. The cells were then grown at the lower temperature of 24 °C and agitated at 180 rpm for 24 h after which time they were centrifuged at 4000×*g* for 10 min. Any remaining growth media was removed by exchanging with 100 mM phosphate buffer pH 8.5. Finally, the resulting pellet was stored at − 20 °C until use.

### C-terminal truncations of human FMO3

The previously published hydropathy plot of the amino acid sequence of hFMO3 [24] had identified the C-terminus as a membrane anchor and consequently three C-terminally truncated hFMO3 constructs (516X, 505X and 493X) were generated in our lab [24]. In the current work, these 3 proteins were heterologously expressed in *E. coli* JM109 in the same way as the full-length protein in order to evaluate whether any of these truncated proteins (higher cytosolic versus membrane-bound) could improve the conversion yields of the substrates in whole-cell catalysis.

### Chemicals tested for regeneration of NADPH

Human FMO3 is an NADPH-dependent enzyme and therefore due to the high price of this cofactor it is vital that a regeneration system is set up for preparative applications. The previously reported NADPH regenerating system NADP^+^/citrate/MgCl_2_ [20] was therefore investigated and the effects of the addition of each of the latter compounds, alone and/or in combination, on NADPH regeneration was monitored at 340 nm. Moreover, the simple addition of glucose (50 mM) was also tested. The amount of NADPH produced in each case was determined using the molar extinction coefficient value of 6.22 mM^−1^cm^−1^.

### Effect of various organic solvents

Ten organic solvents with different polarity index were selected in order to test whether any of them would result in an increase in conversion yields of tamoxifen. A typical reaction mixture contained the recombinant cells (OD_600_ of 10), 2 mM tamoxifen, 0.3 mM NADP^+^, 50 mM tri-sodium citrate, 10 mM MgCl_2_ in 100 mM phosphate buffer pH 8.5 with 10% (v/v) organic solvent in a total volume of 8 mL. The reactions were carried out at 37 °C, 200 rpm for 5 h after which time they were terminated by the addition of ice-cold acetonitrile followed by HPLC analysis. Control experiments were performed in the absence of organic solvent using phosphate buffer pH 8.5.

### HPLC analyses of metabolites

For each of the FMO3-mediated whole-cell catalyses, cell pellet (OD_600_ = 10) was resuspended in 8 mL of 100 mM phosphate buffer pH 8.5 (filter-sterilized). The reaction was then initiated by mixing of the test substrates (1 mM final concentration), NADP^+^ (0.3 mM final concentration), tri-sodium citrate (50 mM final concentration), MgCl_2_ (10 mM final concentration) to the cell suspension. The reaction mixture was incubated at 37 °C, 200 rpm. 200 μL samples were drawn at certain time points, mixed with 200 μL acetonitrile and centrifuged for 5 min at 16,300×*g*. The resulting supernatant was subsequently analysed by HPLC (Agilent-1200, Agilent Technologies, USA) equipped with 4.6 × 150 mm 5 μm Eclipse XDB-C18 column, at 22 °C.

Two different controls were carried out to confirm that the N-oxide metabolites were indeed the result of hFMO3 catalysis. In the first control, non-transformed *E. coli* JM109 were used in the presence of tamoxifen as the substrate. The second control was carried out in order to confirm that the cells were intact and there was no enzyme leakage. In this case, the pellet (transformed *E. coli* JM109-hFMO3) was resuspended in potassium phosphate buffer pH 8.5 and centrifuged at 16,300×*g* for 5 min, the resulting supernatant was used for the enzymatic reaction again in the presence of tamoxifen as described above.

In order to systematically analyse the products of the whole-cell reactions the same mobile phase of acetonitrile (A) and 0.1% formic acid (B) in water was used for the separation of all products from their corresponding parent drug. In all cases an isocratic elution was performed for the HPLC analyses. This meant that only the ratios of the two different mobile phases were modified for each of the 4 drugs; clomiphene (34% A and 66% B, monitored at 290 nm) [16], dasatinib (22% A and 78% B, monitored at 320 nm), GSK5182 (25% A and 75% B, monitored at 280 nm), and tozasertib (20% A and 80% B, monitored at 250 nm). Tamoxifen which was used for in the organic solvent as well as the truncated human FMO3 experiments, was also separated from its N-oxide product using the same mobile phases (40% A and 60% B and monitored at 276 nm). All measurements were performed in triplicates.

N-oxide metabolites of GSK5182 and tozasertib are not commercially available however, these metabolites have been previously identified and confirmed by mass spectrometry analysis by our group [16, 18].

### Identification of dasatinib N-oxide by LC–MS

The identification of the dasatinib N-oxide product was performed in collaboration with Ion Source & Biotechnologies Srl (Milan, Italy) as reported previously for the GSK5182 using an Orbitrap mass spectrometer (ThermoFisher, San Jose, USA) working in positive ion mode [16]. Spectra acquisition was conducted in the 40–3500 m/z range. ESI capillary voltage was kept at 1500 V, SACI surface voltage was set to 47 V, drying gas: 2 L/min, nebulizer gas at 80 psi and temperature at 40 °C. Fragment and precursor ions were isolated through an ion trap and in order to obtain the fragments with high accurate m/z ratio (resolution 15,000, m/z error < 10 ppm) the Orbitrap mass analyser was used.

## Supplementary information

**Additional file 1: Table S1.** Effect of organic solvents (10% v/v) on whole-cell-mediated biotransformation of 2 mM tamoxifen (5-h incubation at 37 °C) compared to control (phosphate buffer pH 8.5). **Fig. S1.** Fragmentation profile of dasatinib N-oxide. Enzymatic N-oxide product with the molecular ion m/z 504.1 (A) and subsequent result of the cleavage of 387.1, 461.1 and 460.2 in MS (B).

## Data Availability

All data generated or analysed during this study are included in this published article and its additional files.

## Abbreviations

FMO3: Flavin-containing monooxygenase isoform 3
GSK5182: (4-[1-[4-[2-(Dimethylamino)ethoxy]phenyl]-5-hydroxy-2-phenylpent-1-enyl]phenol)
HPLC: High performance liquid chromatography
IPTG: Isopropyl-β-d-thiogalactoside
MS: Mass spectrometry
NADP^+^: Nicotinamide adenine dinucleotide phosphate, oxidized form
NADPH: Nicotinamide adenine dinucleotide phosphate
TB: Terrific broth

## References

[1] Cashman JR (2005). Some distinctions between flavin-containing and cytochrome P450 monooxygenases. Biochem Biophys Res Commun.

[2] Krueger SK, Williams DE (2005). Mammalian flavin-containing monooxygenases: structure/function, genetic polymorphisms and role in drug metabolism. Pharmacol Ther.

[3] Phillips IR, Shephard EA (2019). Endogenous roles of mammalian flavin-containing monooxygenases. Catalysts..

[4] Cusack KP, Koolman HF, Lange UEW, Peltier HM, Piel I, Vasudevan A (2013). Emerging technologies for metabolite generation and structural diversification. Bioorg Med Chem Lett.

[5] Geier M, Bachler T, Hanlon SP, Eggimann FK, Kittelmann M, Weber H, Lütz S, Wirz B, Winkler M (2015). Human FMO2-based microbial whole-cell catalysts for drug metabolite synthesis. Microb Cell Fact.

[6] Schroer K, Kittelmann M, Lütz S (2010). Recombinant human cytochrome P450 monooxygenases for drug metabolite synthesis. Biotechnol Bioeng.

[7] Lawton MP, Philpot RM (1993). Functional characterization of Flavin-Containing Monooxygenase 1B1. J Biol Chem.

[8] Sadeghi SJ, Meirinhos R, Catucci G, Dodhia VR, Di Nardo G, Gilardi G (2010). Direct electrochemistry of drug metabolizing human flavin-containing monooxygenase: electrochemical turnover of benzydamine and tamoxifen. J Am Chem Soc.

[9] Catucci G, Sadeghi SJ, Gilardi G (2019). A direct time-based ITC approach for substrate turnover measurements demonstrated on human FMO3. Chem Commun.

[10] Castrignano S, Sadeghi SJ, Gilardi G (2010). Electro-catalysis by immobilized human flavin-containing monooxygenase isoform 3 (hFMO3). Anal Bioanal Chem.

[11] Rostami-Hodjegan A, Lennard MS, Tucker GT, Ledger WL (2004). Monitoring plasma concentrations to individualize treatment with clomiphene citrate. Fertil Steril.

[12] Dewailly D, Hieronimus S, Mirakian P, Hugues JN (2010). Polycystic ovary syndrome (PCOS). Annales D Endocrinologie.

[13] Catucci G, Polignano I, Cusumano D, Medana C, Gilardi G, Sadeghi SJ (2017). Identification of human flavin-containing monooxygenase 3 substrates by a colorimetric screening assay. Anal Biochem.

[14] Kamath AV, Wang J, Lee FY, Marathe PH (2007). Preclinical pharmacokinetics and in vitro metabolism of dasatinib (BMS-354825): a potent oral multi-targeted kinase inhibitor against SRC and BCR-ABL. Cancer Chemoth Pharma..

[15] Joo J, Wu Z, Lee B, Shon JC, Lee T, Lee I-K, Sim T, Kim K-H, Kim ND, Kim SH, Liu K-H (2015). In vitro metabolism of an estrogen-related receptor γ modulator, GSK5182, by human liver microsomes and recombinant cytochrome P450s. Biopharm Drug Dispos.

[16] Catucci G, Bortolussi S, Rampolla G, Cusumano D, Gilardi G, Sadeghi SJ (2018). Flavin-containing monooxygenase 3 polymorphic variants significantly affect clearance of tamoxifen and clomiphene. Basic Clin Pharmacol Toxicol.

[17] Ballard JE, Prueksaritanont T, Tang C (2007). Hepatic Metabolism of MK-0457, a potent aurora kinase inhibitor: interspecies comparison and role of human cytochrome P450 and flavin-containing monooxygenase. Drug Metab Dispos.

[18] Catucci G, Occhipinti A, Maffei M, Gilardi G, Sadeghi S (2013). Effect of human flavin-containing monooxygenase 3 polymorphism on the metabolism of aurora kinase inhibitors. Int J Mol Sci.

[19] Duetz WA, Beilen JBV, Witholt B (2001). Using proteins in their natural environment: potential and limitations of microbial whole-cell hydroxylations in applied biocatalysis. Curr Opin Biotechnol.

[20] Hanlon SP, Camattari A, Abad S, Glieder A, Kittelmann M, Lütz S, Wirz B, Winkler M (2012). Expression of recombinant human flavin monooxygenase and moclobemide-N-oxide synthesis on multi-mg scale. Chem Commun.

[21] Liu Z-Q, Wu L, Zheng L, Wang W-Z, Zhang X-J, Jin L-Q, Zheng Y-G (2018). Biosynthesis of tert -butyl (3 R,5 S)-6-chloro-3,5-dihydroxyhexanoate by carbonyl reductase from *Rhodosporidium toruloides* in mono and biphasic media. Bioresour Technol.

[22] Krueger SK, VanDyke JE, Williams DE, Hines RN (2006). The role of flavin-containing monooxygenase (FMO) in the metabolism of tamoxifen and other tertiary amines. Drug Metab Rev.

[23] Schlegal S, Klepsch M, Gialama D, Wickstrom D, Slotboom DJ, De Gier J-W (2010). Revolutionizing membrane protein overexpression in bacteria. Microb Biotechnol.

[24] Catucci G, Gilardi G, Jeuken L, Sadeghi SJ (2012). In vitro drug metabolism by C-terminally truncated human flavin-containing monooxygenase 3. Biochem Pharmacol.

[25] Castrignanò S, Gilardi G, Sadeghi SJ (2015). Human flavin-containing monooxygenase 3 on graphene oxide for drug metabolism screening. Anal Chem.

