# Supplementary material for: Production of drug metabolites by human FMO3 in Escherichia coli
Source: Microb Cell Fact. 2020 Mar 20;19:74. doi: 10.1186/s12934-020-01332-1 (PMC7085137; doi:10.1186/s12934-020-01332-1)
Supplement: Supplementary file 1 — Additional file 1: Table S1. Effect of organic solvents (10% v/v) on whole-cell-mediated biotransformation of 2 mM tamoxifen (5-h incubation at 37 °C) compared to control (phosphate buffer pH 8.5). Fig. S1. Fragmentation profile of dasatinib N-oxide. Enzymatic N-oxide product with the molecular ion m/z 504.1 (A) and subsequent result of the cleavage of 387.1, 461.1 and 460.2 in MS (B). [file 12934_2020_1332_MOESM1_ESM.docx]

**Additional file**

**Production of drug metabolites by FMO3 in *E. coli***

[Gianluca Catucci](http://www.sciencedirect.com/science/article/pii/S0003269717300465), [Gianfranco Gilardi](http://www.sciencedirect.com/science/article/pii/S0003269717300465), [Sheila J. Sadeghi](http://www.sciencedirect.com/science/article/pii/S0003269717300465)*****

Department of Life Sciences and Systems Biology, University of Torino, Torino, Italy

* Corresponding author at: Department of Life Sciences and Systems Biology, Via Accademia Albertina 13, 10123 Torino, Italy.

Tel.: +39 011 6704528;

Fax: +39 011 6704643.

E-mail address: sheila.sadeghi@unito.it (S.J. Sadeghi).

|  | Conversion Yield (%) | Solvent polarity index^1^ |
| --- | --- | --- |
| Control | 51 | n/a |
| Ethyl acetate | 6.8 | 0.228 |
| Chloroform | 1.5 | 0.259 |
| Acetone | 7 | 0.355 |
| DMF | 6.5 | 0.386 |
| DMSO | 6 | 0.444 |
| 2-propanol | 10 | 0.546 |
| Acetyl acetone | 1.8 | 0.571 |
| 1-butanol | 2.5 | 0.586 |
| Ethanol | 9 | 0.654 |
| Methanol | 25 | 0.762 |

^1^Solvents and Solvent Effects in Organic Chemistry, Wiley-VCH Publishers, 3rd ed., 2003.

**Table S1** Effect of organic solvents (10% v/v) on whole-cell-mediated biotransformation of 2 mM tamoxifen (5-hour incubation at 37 °C) compared to control (phosphate buffer pH 8.5).

**A**


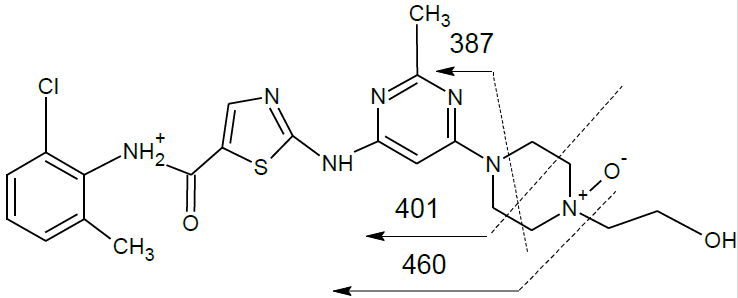


B


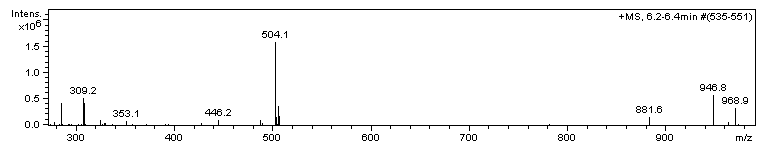


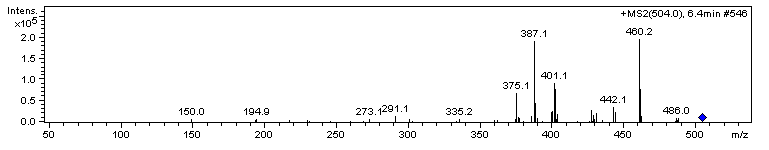


**Fig. S1** Fragmentation profile of dasatinib N-oxide. Enzymatic N-oxide product with the molecular ion m/z 504.1 (A) and subsequent result of the cleavage of 387.1, 461.1 and 460.2 in MS (B).
